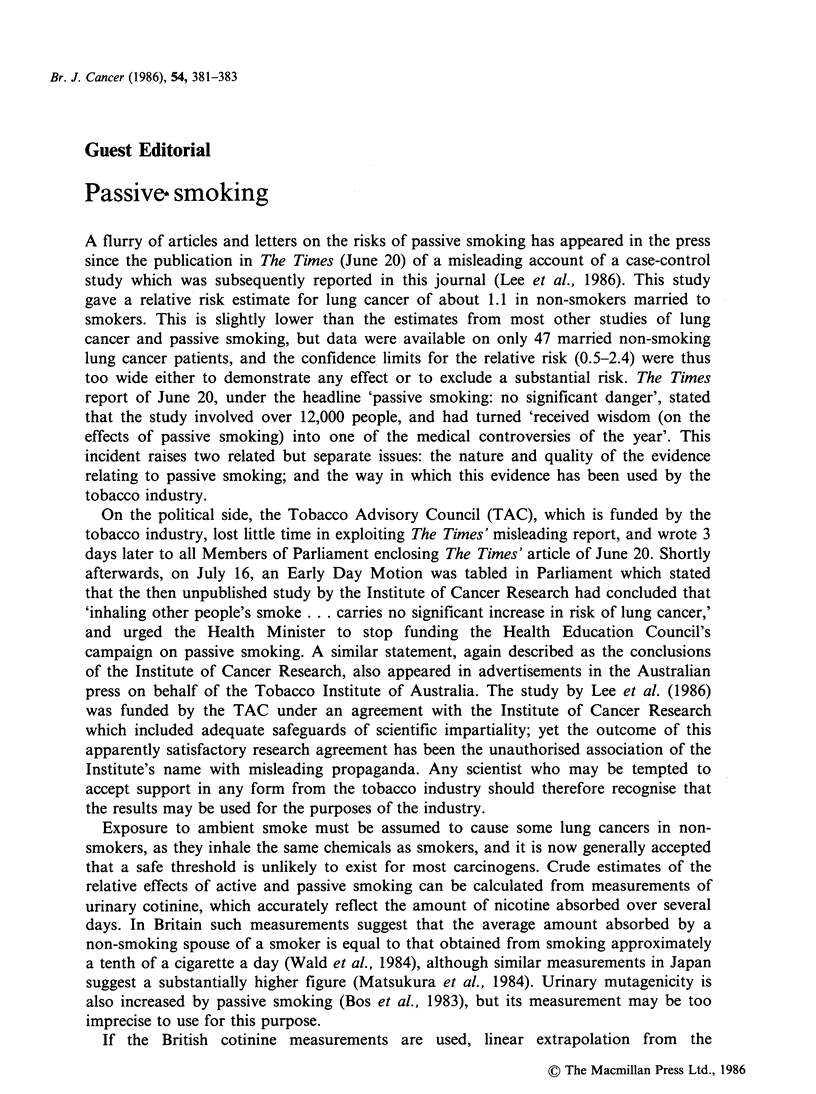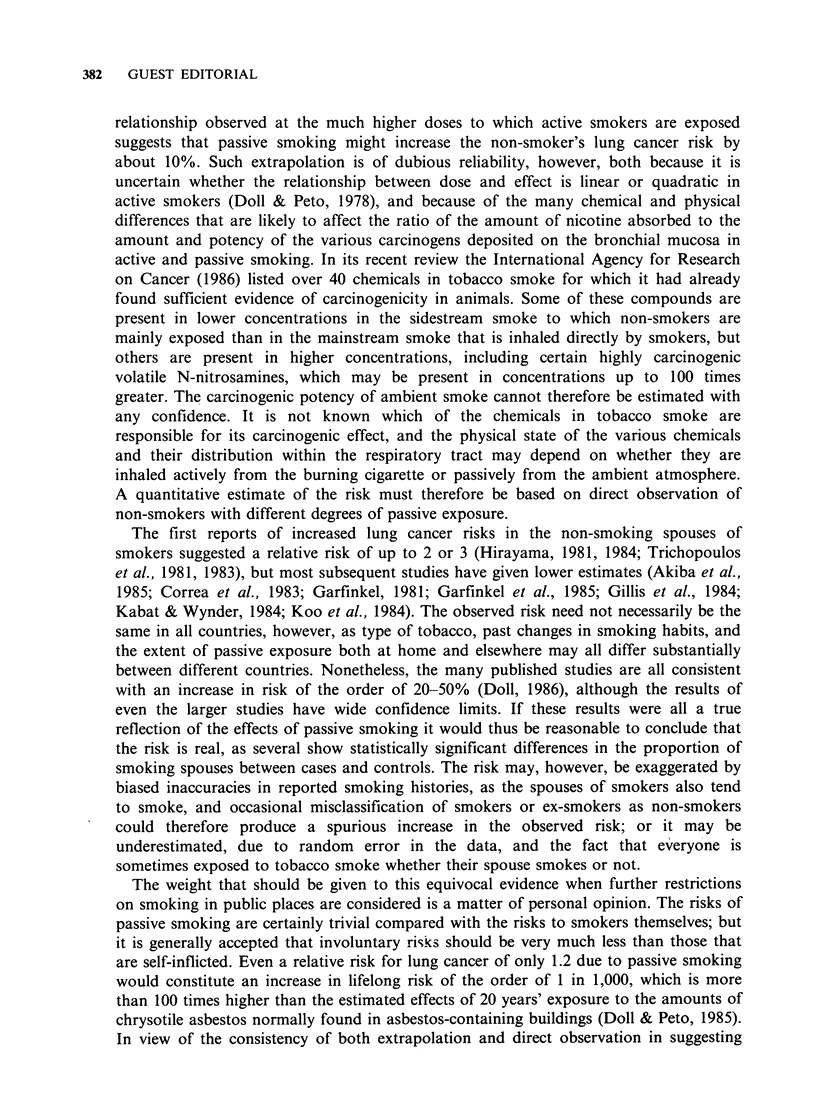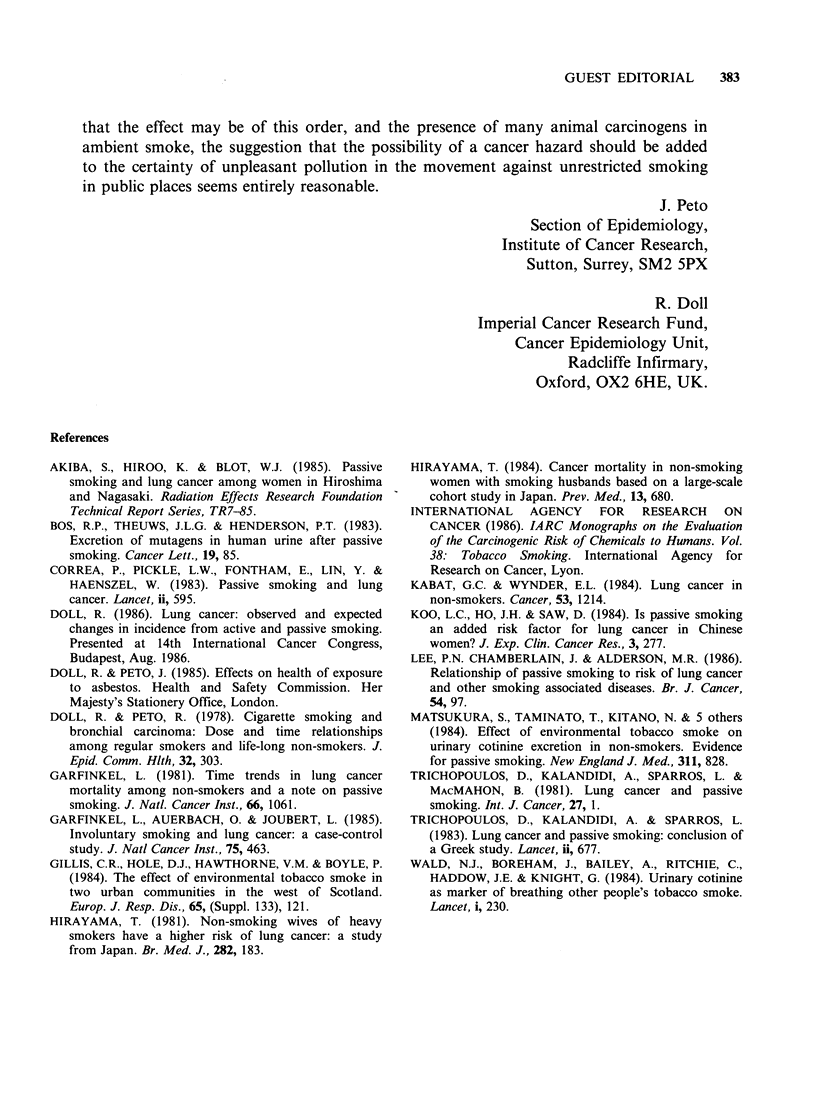# Passive smoking.

**DOI:** 10.1038/bjc.1986.187

**Published:** 1986-09

**Authors:** J. Peto, R. Doll


*Br. J. Cancer* (1986), **54**, 381–383

### Guest Editorial

### Passive smoking

A flurry of articles and letters on the risks of passive smoking has appeared in the press since the publication in *The Times* (June 20) of a misleading account of a case-control study which was subsequently reported in this journal (Lee *et al.,* 1986). This study gave a relative risk estimate for lung cancer of about 1.1 in non-smokers married to smokers. This is slightly lower than the estimates from most other studies of lung cancer and passive smoking, but data were available on only 47 married non-smoking lung cancer patients, and the confidence limits for the relative risk (0.5–2.4) were thus too wide either to demonstrate any effect or to exclude a substantial risk. *The Times* report of June 20, under the headline 'passive smoking: no significant danger', stated that the study involved over 12,000 people, and had turned 'received wisdom (on the effects of passive smoking) into one of the medical controversies of the year'. This incident raises two related but separate issues: the nature and quality of the evidence relating to passive smoking; and the way in which this evidence has been used by the tobacco industry.

On the political side, the Tobacco Advisory Council (TAC), which is funded by the tobacco industry, lost little time in exploiting *The Times'* misleading report, and wrote 3 days later to all Members of Parliament enclosing *The Times'* article of June 20. Shortly afterwards, on July 16, an Early Day Motion was tabled in Parliament which stated that the then unpublished study by the Institute of Cancer Research had concluded that 'inhaling other people's smoke . . . carries no significant increase in risk of lung cancer,' and urged the Health Minister to stop funding the Health Education Council's campaign on passive smoking. A similar statement, again described as the conclusions of the Institute of Cancer Research, also appeared in advertisements in the Australian press on behalf of the Tobacco Institute of Australia. The study by Lee *et al.* (1986) was funded by the TAC under an agreement with the Institute of Cancer Research which included adequate safeguards of scientific impartiality; yet the outcome of this apparently satisfactory research agreement has been the unauthorised association of the Institute's name with misleading propaganda. Any scientist who may be tempted to accept support in any form from the tobacco industry should therefore recognise that the results may be used for the purposes of the industry.

Exposure to ambient smoke must be assumed to cause some lung cancers in non-smokers, as they inhale the same chemicals as smokers, and it is now generally accepted that a safe threshold is unlikely to exist for most carcinogens. Crude estimates of the relative effects of active and passive smoking can be calculated from measurements of urinary cotinine, which accurately reflect the amount of nicotine absorbed over several days. In Britain such measurements suggest that the average amount absorbed by a non-smoking spouse of a smoker is equal to that obtained from smoking approximately a tenth of a cigarette a day (Wald *et al.,* 1984), although similar measurements in Japan suggest a substantially higher figure (Matsukura *et al.,* 1984). Urinary mutagenicity is also increased by passive smoking (Bos *et al.,* 1983), but its measurement may be too imprecise to use for this purpose.

If the British cotinine measurements are used, linear extrapolation from the

© The Macmillan Press Ltd., 1986

relationship observed at the much higher doses to which active smokers are exposed suggests that passive smoking might increase the non-smoker's lung cancer risk by about 10%. Such extrapolation is of dubious reliability, however, both because it is uncertain whether the relationship between dose and effect is linear or quadratic in active smokers (Doll & Peto, 1978), and because of the many chemical and physical differences that are likely to affect the ratio of the amount of nicotine absorbed to the amount and potency of the various carcinogens deposited on the bronchial mucosa in active and passive smoking. In its recent review the International Agency for Research on Cancer (1986) listed over 40 chemicals in tobacco smoke for which it had already found sufficient evidence of carcinogenicity in animals. Some of these compounds are present in lower concentrations in the sidestream smoke to which non-smokers are mainly exposed than in the mainstream smoke that is inhaled directly by smokers, but others are present in higher concentrations, including certain highly carcinogenic volatile N-nitrosamines, which may be present in concentrations up to 100 times greater. The carcinogenic potency of ambient smoke cannot therefore be estimated with any confidence. It is not known which of the chemicals in tobacco smoke are responsible for its carcinogenic effect, and the physical state of the various chemicals and their distribution within the respiratory tract may depend on whether they are inhaled actively from the burning cigarette or passively from the ambient atmosphere. A quantitative estimate of the risk must therefore be based on direct observation of non-smokers with different degrees of passive exposure.

The first reports of increased lung cancer risks in the non-smoking spouses of smokers suggested a relative risk of up to 2 or 3 (Hirayama, 1981, 1984; Trichopoulos *et al.*, 1981, 1983), but most subsequent studies have given lower estimates (Akiba *et al.*, 1985; Correa *et al.*, 1983; Garfinkel, 1981; Garfinkel *et al.*, 1985; Gillis *et al.*, 1984; Kabat & Wynder, 1984; Koo *et al.*, 1984). The observed risk need not necessarily be the same in all countries, however, as type of tobacco, past changes in smoking habits, and the extent of passive exposure both at home and elsewhere may all differ substantially between different countries. Nonetheless, the many published studies are all consistent with an increase in risk of the order of 20–50% (Doll, 1986), although the results of even the larger studies have wide confidence limits. If these results were all a true reflection of the effects of passive smoking it would thus be reasonable to conclude that the risk is real, as several show statistically significant differences in the proportion of smoking spouses between cases and controls. The risk may, however, be exaggerated by biased inaccuracies in reported smoking histories, as the spouses of smokers also tend to smoke, and occasional misclassification of smokers or ex-smokers as non-smokers could therefore produce a spurious increase in the observed risk; or it may be underestimated, due to random error in the data, and the fact that everyone is sometimes exposed to tobacco smoke whether their spouse smokes or not.

The weight that should be given to this equivocal evidence when further restrictions on smoking in public places are considered is a matter of personal opinion. The risks of passive smoking are certainly trivial compared with the risks to smokers themselves; but it is generally accepted that involuntary risks should be very much less than those that are self-inflicted. Even a relative risk for lung cancer of only 1.2 due to passive smoking would constitute an increase in lifelong risk of the order of 1 in 1,000, which is more than 100 times higher than the estimated effects of 20 years' exposure to the amounts of chrysotile asbestos normally found in asbestos-containing buildings (Doll & Peto, 1985). In view of the consistency of both extrapolation and direct observation in suggesting that the effect may be of this order, and the presence of many animal carcinogens in ambient smoke, the suggestion that the possibility of a cancer hazard should be added to the certainty of unpleasant pollution in the movement against unrestricted smoking in public places seems entirely reasonable.

J. Peto
Section of Epidemiology,
Institute of Cancer Research,
Sutton, Surrey, SM2 5PX

R. Doll
Imperial Cancer Research Fund,
Cancer Epidemiology Unit,
Radcliffe Infirmary,
Oxford, OX2 6HE, UK.

**References**

AKIBA, S., HIROO, K. & BLOT, W.J. (1985). Passive smoking and lung cancer among women in Hiroshima and Nagasaki. *Radiation Effects Research Foundation Technical Report Series, TR7–85.*

BOS, R.P., THEUWS, J.L.G. & HENDERSON, P.T. (1983). Excretion of mutagens in human urine after passive smoking. *Cancer Lett.*, **19**, 85.

CORREA, P., PICKLE, L.W., FONTHAM, E., LIN, Y. & HAENSZEL, W. (1983). Passive smoking and lung cancer. *Lancet*, **ii**, 595.

DOLL, R. (1986). Lung cancer: observed and expected changes in incidence from active and passive smoking. Presented at 14th International Cancer Congress, Budapest, Aug. 1986.

DOLL, R. & PETO, J. (1985). Effects on health of exposure to asbestos. Health and Safety Commission. Her Majesty's Stationery Office, London.

DOLL, R. & PETO, R. (1978). Cigarette smoking and bronchial carcinoma: Dose and time relationships among regular smokers and life-long non-smokers. *J. Epid. Comm. Hlth*, **32**, 303.

GARFINKEL, L. (1981). Time trends in lung cancer mortality among non-smokers and a note on passive smoking. *J. Natl. Cancer Inst.*, **66**, 1061.

GARFINKEL, L., AUERBACH, O. & JOUBERT, L. (1985). Involuntary smoking and lung cancer: a case-control study. *J. Natl Cancer Inst.*, **75**, 463.

GILLIS, C.R., HOLE, D.J., HAWTHORNE, V.M. & BOYLE, P. (1984). The effect of environmental tobacco smoke in two urban communities in the west of Scotland. *Europ. J. Resp. Dis.*, **65**, (Suppl. 133), 121.

HIRAYAMA, T. (1981). Non-smoking wives of heavy smokers have a higher risk of lung cancer: a study from Japan. *Br. Med. J.*, **282**, 183.

HIRAYAMA, T. (1984). Cancer mortality in non-smoking women with smoking husbands based on a large-scale cohort study in Japan. *Prev. Med.*, **13**, 680.

INTERNATIONAL AGENCY FOR RESEARCH ON CANCER (1986). *IARC Monographs on the Evaluation of the Carcinogenic Risk of Chemicals to Humans. Vol. 38: Tobacco Smoking.* International Agency for Research on Cancer, Lyon.

KABAT, G.C. & WYNDER, E.L. (1984). Lung cancer in non-smokers. *Cancer*, **53**, 1214.

KOO, L.C., HO, J.H. & SAW, D. (1984). Is passive smoking an added risk factor for lung cancer in Chinese women? *J. Exp. Clin. Cancer Res.*, **3**, 277.

LEE, P.N. CHAMBERLAIN, J. & ALDERSON, M.R. (1986). Relationship of passive smoking to risk of lung cancer and other smoking associated diseases. *Br. J. Cancer*, **54**, 97.

MATSUKURA, S., TAMINATO, T., KITANO, N. & 5 others (1984). Effect of environmental tobacco smoke on urinary cotinine excretion in non-smokers. Evidence for passive smoking. *New England J. Med.*, **311**, 828.

TRICHOPOULOS, D., KALANDIDI, A., SPARROS, L. & MACMAHON, B. (1981). Lung cancer and passive smoking. *Int. J. Cancer*, **27**, 1.

TRICHOPOULOS, D., KALANDIDI, A. & SPARROS, L. (1983). Lung cancer and passive smoking: conclusion of a Greek study. *Lancet*, **ii**, 677.

WALD, N.J., BOREHAM, J., BAILEY, A., RITCHIE, C., HADDOW, J.E. & KNIGHT, G. (1984). Urinary cotinine as marker of breathing other people's tobacco smoke. *Lancet*, **i**, 230.